# The Effect of a BSA Conjugate of a Synthetic Hexasaccharide Related to the Fragment of Capsular Polysaccharide of *Streptococcus pneumoniae* Type 14 on the Activation of Innate and Adaptive Immune Responses

**DOI:** 10.3389/fimmu.2016.00248

**Published:** 2016-06-24

**Authors:** Nelli K. Akhmatova, Ekaterina A. Kurbatova, Elvin A. Akhmatov, Nadezhda B. Egorova, Denis Yu. Logunov, Marina L. Gening, Elena V. Sukhova, Dmitry V. Yashunsky, Yury E. Tsvetkov, Nikolay E. Nifantiev

**Affiliations:** ^1^Department of Immunology, Mechnikov Research Institute for Vaccines and Sera, Russian Academy of Medical Sciences, Moscow, Russia; ^2^Department of Microbiology, Gamaleya Research Institute for Epidemiology and Microbiology, Russian Ministry of Health, Moscow, Russia; ^3^Department of Glycoconjugate Chemistry, N. D. Zelinsky Institute of Organic Chemistry, Russian Academy of Sciences, Moscow, Russia

**Keywords:** synthetic hexasaccharide conjugate, dendritic cell, cytokine, antibody, cell-surface molecule

## Abstract

We report the effect of a bovine serum albumin (BSA) conjugate of a synthetic hexasaccharide (HS) related to the fragment of the capsular polysaccharide (PS) of *Streptococcus pneumoniae* type 14 on the stimulation of innate immune system and the subsequent development of a PS-specific antibody response. Glycoconjugate (GC) in the presence (GC + AL) or absence of aluminum hydroxide was administered to mice twice. GC increased the number of TLR2-expressing cells and induced the maturation of dendritic cells (CD11c^+^, CD80^+^ and, MHCII^+^), which secreted IL-1β, IL-6, and TNFα into the culture medium. The level of IL-1β, IL-10, IFNγ, and TNFα in the blood increased within 24 h after the single GC administration to mice. On day 7, the numbers of splenic CD4^+^ and CD8^+^ T lymphocytes and B lymphocytes increased. After the second immunization, the levels of CD4^+^ and CD8^+^ T lymphocytes were lower than in the control, whereas the B cell, NK cell, and MHC class II-expressing cell numbers remained enhanced. However, of the presence of anti-PS, IgG antibodies were not detected. The addition of aluminum hydroxide to GC stimulated the production of GM-CSF, IL-1β, IL-5, IL-6, IL-10, IL-17, IFNγ, and TNFα. Anti-PS IgG1 antibody titers 7 days after the second immunization were high. During that period, normal levels of splenic CD4^+^ T lymphocytes were maintained, whereas reduced CD8^+^ T lymphocyte numbers and increased levels of B lymphocytes, NK cells, and MHC class II-expressing cell numbers were observed. Anti-PS IgG levels diminished until day 92. A booster immunization with GC + AL stimulated the production of anti-PS IgG memory antibodies, which were determined within 97 days. The elucidation of specific features of the effect of the synthetic HS conjugate on the stimulation of innate, cell-mediated immunity, and antibody response can favor the optimization of GC vaccine design.

## Introduction

*Streptococcus pneumoniae* is one of the major etiological factors of bacterial pneumonia, bacteremia, meningitis, otitis media, and other serious pneumococcal childhood and adult diseases (1–5) and has a high death rate (6–8). A large number of pneumococcal strains are surrounded by a polysaccharide (PS) capsule. According to the chemical structure of the capsule, more than 90 serotypes of *S. pneumoniae* were identified (5, 9). Among them, 20 serotypes of *S. pneumoniae* cause approximately 80% of diseases (7, 10, 11).

IgG antibodies to capsular PS *S. pneumoniae* (12) and complement-mediated opsonophagocytosis (13–15) have been found to be the main effector mechanisms underlying protection from pneumococcal infection.

Capsular pneumococcal PSs and oligosaccharides are classified as T-independent antigens, which induce the formation of predominantly low-affinity IgM antibodies (12). One of the mechanisms for the induction of the T-dependent IgG immune response to capsular PSs or oligosaccharides is their conjugation with a carrier protein (12, 16–18). Some oligosaccharides that are conjugated with protein induce the formation of antibodies to capsular PSs at a higher level than that of traditional PS conjugate vaccines (19).

The mechanism of the development of the immune response to T-dependent antigens is presently well characterized (12, 20). In contrast, the activation of innate and adaptive immunity under the action of glycoconjugates (GCs), consisting of a carbohydrate part to which the induction of protective IgG antibodies occurs and a T-dependent protein carrier covalently bound to the carbohydrate part, remains insufficiently studied. Recently obtained data indicate that after processing, the GC in antigen-presenting cells (APCs), the peptide component is bound to MHC class II and the glycan, is recognized by unique carbohydrate-specific CD4^+^ T cells (21).

It is known that capsular carbohydrates interact with B cells through the B cell receptor (BCR), resulting in the proliferation and differentiation of B lymphocytes (12). PSs affect dendritic cells (DCs) through the specific intracellular adhesion molecule-3 grabbing non-integrin (SIGN) receptor or the mannose receptor (C-type lectin) (22). The data on the activation of toll-like receptors (TLRs) under the action of bacterial capsular PS are ambiguous (23, 24). According to Meltzer and Goldblatt, DCs did not mature and produced no cytokines under the action of bacterial capsular PSs from *S. pneumoniae* of serotypes 1, 6B, 9N, 14, 19F, or 23F (24).

The capsular PS of *S. pneumoniae* serotype 14 consists of branched tetrasaccharide repeated units (25) and is weakly immunogenic compared to the capsular PSs of other pneumococcal serotypes (26, 27). Most of the investigations were devoted to the study of the immunogenic properties of tetrasaccharide, which is considered to be the main candidate for inclusion into a synthetic multivalent pneumococcal vaccine (28).

The activation of the innate immune system in response to protein conjugates of synthetic oligosaccharides has never before been studied. It is an important stage for the analysis of immunological properties toward the development of a vaccine because innate immunity determines the direction, length, and effectiveness of the adaptive immune response. For these studies, it was necessary to choose the appropriate oligosaccharide that met special requirements. The oligosaccharide, as a part of the protein conjugate, was required to be immunogenic to allow the analysis of the influence of the main innate immune factors on the production of specific antibodies and other factors of adaptive immunity. In this study, immunologically active branched hexasaccharide (HS) β-d-Gal-(1 → 4)-β-d-Glc-(1 → 6)-[β-d-Gal-(1 → 4)]-β-d-GlcNAc-(1 → 3)-β-d-Gal-(1 → 4)-β-d-Glc was chosen (28). It is important to note that HS not only represents one repeating unit of PS *S. pneumoniae* type 14 but also includes two additional monosaccharides. This structural fragment bears greater similarities to PS *S. pneumoniae* type 14 compared to shorter oligosaccharides and therefore, being a part of the GC, initiates an immune response that is closer to the action of the PS and conjugated pneumococcal vaccines.

In this work, we studied the effect of a synthetic HS representing a region of the chain of capsular PS *S. pneumoniae* serotype 14 conjugated with BSA. The clinical significance of this *S. pneumoniae* serotype, especially for children, was confirmed by epidemiological studies (5, 29).

For the first time, a collection of data is presented on the effect of this HS and its conjugate with BSA on the *in vitro* and *in vivo* activation of TLRs, the maturation of DCs, and the production of cytokines in mice immunized with the hexacaccharide conjugate both absorbed and unabsorbed on aluminum hydroxide. The effect of the GC on the change in the splenic mononuclear leukocyte phenotype of immunized mice and its ability to induce the production of antibodies to capsular PS is also shown here.

## Materials and Methods

### Synthetic Hexasaccharide-BSA Conjugate

**Figure 1:**
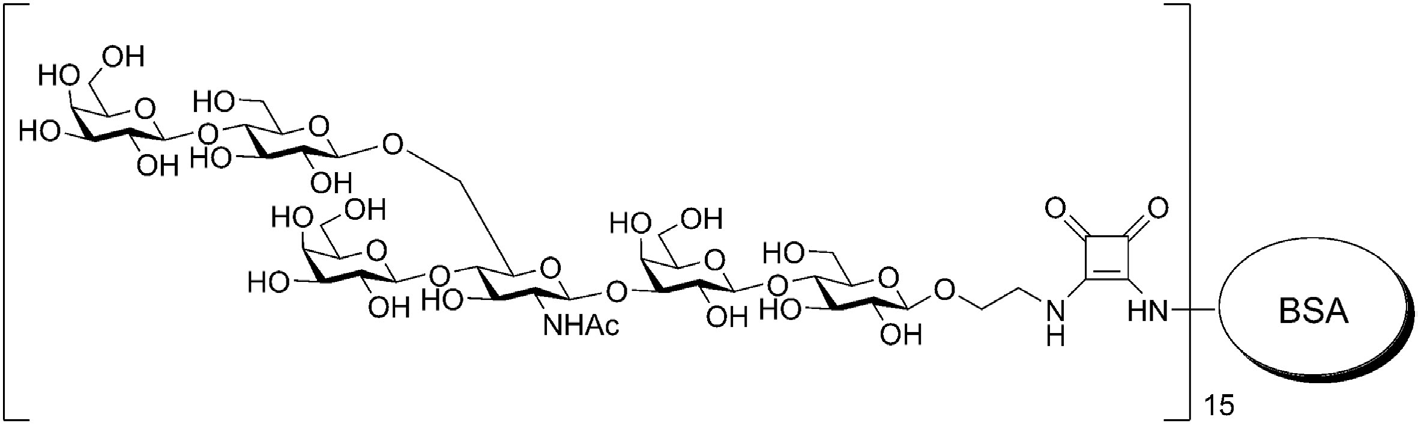
**Chemical structure of studied glycoconjugate immunogen**.

The HS conjugate [β-Gal-(1 → 4)-β-Glc-(1 → 6)-[β-Gal-(1 → 4)]-β-GlcNAc-(1 → 3)-β-Gal-(1 → 4)-β-Glc-O-spacer]_15_-BSA was prepared by the squarate method and contained, according to MALDI-TOF data, on average, 15 HS residues as previously described (30, 31) and 19% carbohydrates on a dry weight basis (Figure 1). The BSA is often used as a protein carrier to design immunogenic GCs and biomolecular systems (32) of other types. For conjugation with HS used BSA (Sigma, USA).

The capsular PS was obtained from the laboratory strain of *S. pneumoniae* type 14 #883 isolated April 22, 2012 from a child with acute otitis media. The strain was grown in a semisynthetic nutrient medium. The isolation of PS included ultrafiltration and concentration, an enzyme treatment, phenolic deproteinization, and dialysis. Resulting preparations contained <5% of denatured proteins and <1% of nucleic acids, while the major portion was carbohydrates. The specificity of PS tested in Rocket Immunoelectrophoresis and inhibition ELISA, where PS gave the rocket with the hyperimmune rabbit serum to *S. pneumoniae* type 14 (in a dose of 1 μg/mL) caused 100% inhibition of the ELISA reaction with the same serum at a dilution of 1:6400–1:12800. The presence of PS in the preparation was confirmed by NMR spectrometry.

### Animals

BALB/c male mice (*n* = 30) were used for evaluation of the antibody production and CBA female mice (*n* = 96) were used for determination of cell surface molecular expression and cytokine production. Mice aged 6–8 weeks were purchased from the Scientific and Production Centre for Biomedical Technologies, Branch “Andreevka” (Moscow, Russia) and kept in the vivarium of the Mechnikov Research Institute for Vaccines and Sera. The housing, husbandry, blood sampling, and sacrifice conditions conformed to the European Union guidelines for the care and use of laboratory animals. The design of experiments was approved by the Ethics Committee of Mechnikov Research Institute for Vaccines and Sera.

### Immunization

Mice were immunized intraperitoneally twice (on day 0 and 14) with GC in the presence (GC + AL) or absence of aluminum hydroxide (Sigma-Aldrich Co., USA), which served as an adjuvant. The single dose of GCs diluted in saline was 10 μg (calculated with the reference to carbohydrate). Aluminum hydroxide was added in an amount of 25 μL (250 μg) per immunizing dose and stored overnight at +4°C. A booster immunization was administered on day 92 in the same manner and at the same dose of GC with aluminum hydroxide.

### TLRs Activation and Expression

The expression of TLRs was determined by two methods. First, the direct ligand–receptor interaction of the HS, the GC and BSA was estimated with THP1-XBlue™-CD14 cells (Invivogen, USA) derived from the THP1-XBlue™ cell line of human monocytes expressing different pathogen-recognizing receptors, including TLRs, and NOD-like receptors (NLRs). THP1-XBlue™-CD14 cells stably express an NF-κB/AP-1-inducible secreted embryonic alkaline phosphatase (SEAP)-reporter gene and human CD14 gene. TLRs stimulation in THP1-XBlue™-CD14 cells induces signaling cascades leading to the activation of NF-κB and AP-1 and the subsequent production of SEAP. The reporter protein is easily detectable and measurable when using QUANTI-Blue™, a medium that turns purple/blue in the presence of SEAP.

Hexasaccharide conjugated with BSA (GC), HS, and BSA at concentrations of 100 μg/mL were used to estimate SEAP activity. The negative control (C^−^) was phosphate buffered saline (PBS). The following substances served as positive controls (C^+^): lipopolysaccharide (LPS) (ligand for TLR4) (Sigma-Aldrich Co., USA); CBLB502, denoted as Entolimod (Cleveland Biolabs, Inc.), an engineered derivative of a *Salmonella* flagellin protein that induces a spectrum of protective effects upon direct interaction and signaling *via* TLR5; and C12-iE-DAP (Invivogen, USA), an acylated derivative of the dipeptide iE-DAP (γ-d-Glu-mDAP) that is present in the peptidoglycan of Gram-negative bacilli and, particularly, Gram-positive bacteria, and is recognized by the intracellular sensor NOD1, which results in NF-κB activation and the production of inflammatory cytokines. The positive controls were used at a concentration of 1 μg/mL.

Second, the number of TLR2- and TLR4-expressing spleen mononuclear cells was determined by flow cytometry (Cytomix FC-500, Beckman Coulter, USA with the CXP software) using FITC anti-CD282 and PE anti-CD284 monoclonal antibodies (eBioscience, USA), respectively. As the cut-off for the positive expression was the number of TLR2- and TLR4-positive spleen mononuclear cells in non-immunized mice.

### Cell Surface Molecule Expression on Dendritic Cells

Dendritic cells were obtained from mouse bone marrow and cultivated in RPMI-1640 with 20 ng/mL granulocyte-macrophage colony-stimulating factor (GM-CSF) and 20 ng/mL of interleukine-4 (IL-4). On day 7, GC (50 μg/mL) was added to the culture medium. TNFα at a concentration of 20 ng/mL was used as a positive control. Three days after incubation, the DC cell surface molecules and cytokine production were determined by flow cytometry (Cytomix FC-500, Beckman Coulter, USA with the CXP software). The cell population gate was determined by forward and side scattering and cell size; 10,000 cells per gate were recorded. The Abs that was used included: FITC or PE-conjugated Abs against CD34 (clone RAM34, FITC), CD11c (clone HL3, FITC), CD83 (clone Michel-17, FITC), CD86 (clone PO3.1, PE), CD80 (clone 16-10A1, FITC), MHCII (I-EK, PE) (clone 14-4-45); all were obtained from eBioscience (eBioscience Inc., San Diego, CA, USA).

### Cell Surface Molecule Expression on Splenic Mononuclear Cells

Splenocytes were isolated from mice vaccinated with the glyconjugate in the absence or in the presence of aluminum hydroxide 7 days after the first and second immunizations. Single-cell suspensions of splenocytes were prepared by mashing the spleen with the plunger of a disposable syringe, passing the ground spleen through a nylon mesh, and suspending the cells in PBS. Splenic single-cell suspensions were stained with phycoerythrin (PE)- or fluorescein isothiocyanate (FITC)-conjugated anti-mouse antibodies to: CD3, CD4, CD8, CD19, CD5.2, NK1.1, CD25, CD4/CD25 (Treg), TCR (T gamma-delta), and I-EK (MHC class II) monoclonal antibodies (eBioscience, USA) at 4°C for 30 min. Erythrocytes were then lysed with red blood cell lysis buffer (BioLegend, USA). After washing with PBS, the samples were fixed with a fixation solution (BioLegend, USA) and analyzed by flow cytometry (Cytomix FC-500, Beckman Coulter, USA with the CXP software). The cell population gate was determined by forward and side scattering and cell size; 10,000 cells per gate were recorded. Spleen cell suspensions were stained using antibodies specific for mouse CD3e-FITC (clone 145-2C11), CD4-FITC (clone GK1.5), CD8a-FITC (clone 53-6.7), γδT (clone γδ TCR-PE, eBioGL3), CD19-FITC (eBio1D3), CD5-PE (clone 53-7.3) NK.1.1 (clone PK136) CD25-PE (PC61.5), and MHCII-PE (I-EK) (clone 14-4-45) obtained from eBioscience or Biolegend (San Diego, CA, USA). Treg: Alexa Fluor 405-conjugated anti-mouse CD4 (clone RM4-5) was obtained from Caltag Laboratories. Staining with anti-FoxP3-FITC conjugated Ab (clone FJK-16s) was performed according to the manufacturer’s protocol (eBioscience).

### Cytokine Production

The level of cytokines in DC supernatants and in mouse serum obtained at 2, 4, 8, and 24 h after the intraperitoneal injection of GC or GC + AL was determined by the FlowCytomix Mouse Thl/Th2 10-plex Kit test system containing beads coated with monoclonal antibodies to cytokines [GM-CSF, IFNγ, IL-1β, IL-2, IL-4, IL-5, IL-6, IL-10, IL-17, and TNFα (eBioscience, USA)] using a flow cytometric assay (FC-500, Beckman Coulter, USA).

### Antibodies to Pneumococcal PS

Serum PS-specific IgG was detected by ELISA. Blood serum was obtained 7, 14, 21, and 28 days after intraperitoneal immunization of mice with GC and 7, 14, 21, 28, 61, 92, 93, 96, 99, and 189 days after GC + AL administration. Blood samples from six mice were studied for each time point. A booster immunization of GC + AL was carried out on d 92. Anti-PS IgG and subisotype (IgG1, IgG2a, IgG2b, IgG3) levels were determined by the enzyme-linked immunosorbent assay (ELISA) as previously described (30). Specifically, flat-bottom plates (Biochemicals, Russia) were coated with a PS preparation from *S. pneumoniae* type 14 (1 μg/well). The presence of the PS in the preparation was demonstrated by two-dimensional Nuclear Magnetic Resonance (NMR). For secondary antibodies to total IgG, horseradish peroxidase-labeled dry diagnostic antibodies to IgG (H + L) of white mice (Medgamal, Russia) and rabbit anti-mouse peroxidase conjugated IgG1 (gamma 1 chain), IgG2a (gamma 2a chain), IgG 2b (gamma 2b chain), and IgG3 (gamma 3 chain) (Rockland Immunochemicals, Inc., Gilbertsville PA, USA) were used. The optical density (OD) was determined using an ELISA reader (iMark, Japan) at 450 nm. The antibody titers were defined as the dilution of serum giving twice the OD_450_ for control mice (non-immunized) with a cutoff value of 0.2.

### Statistical Analysis

Multiple comparisons of the serum titration data were performed by one-way ANOVA for independent samples, which were Tukey posttested. Log_10_-transformed data of titers were used in all analyses. The groups that failed normality tests were compared using the Mann–Whitney rank sum test for independent samples. A *P* value of ≤0.05 was considered statistically significant (STATISTICA 8 software).

## Results

### Toll-Like Receptor 2 Expression

The action of synthetic hexasaccharide (HS) on pathogen-recognition cell receptors of the innate immune system was evaluated *in vitro* by the level of SEAP by THP1-XBlue-CD14 cells expressing different TLRs and NLRs. In the SEAP assay test, HS, GC, and BSA were used at the same concentration (100 μg/mL).

**Figure 2:**
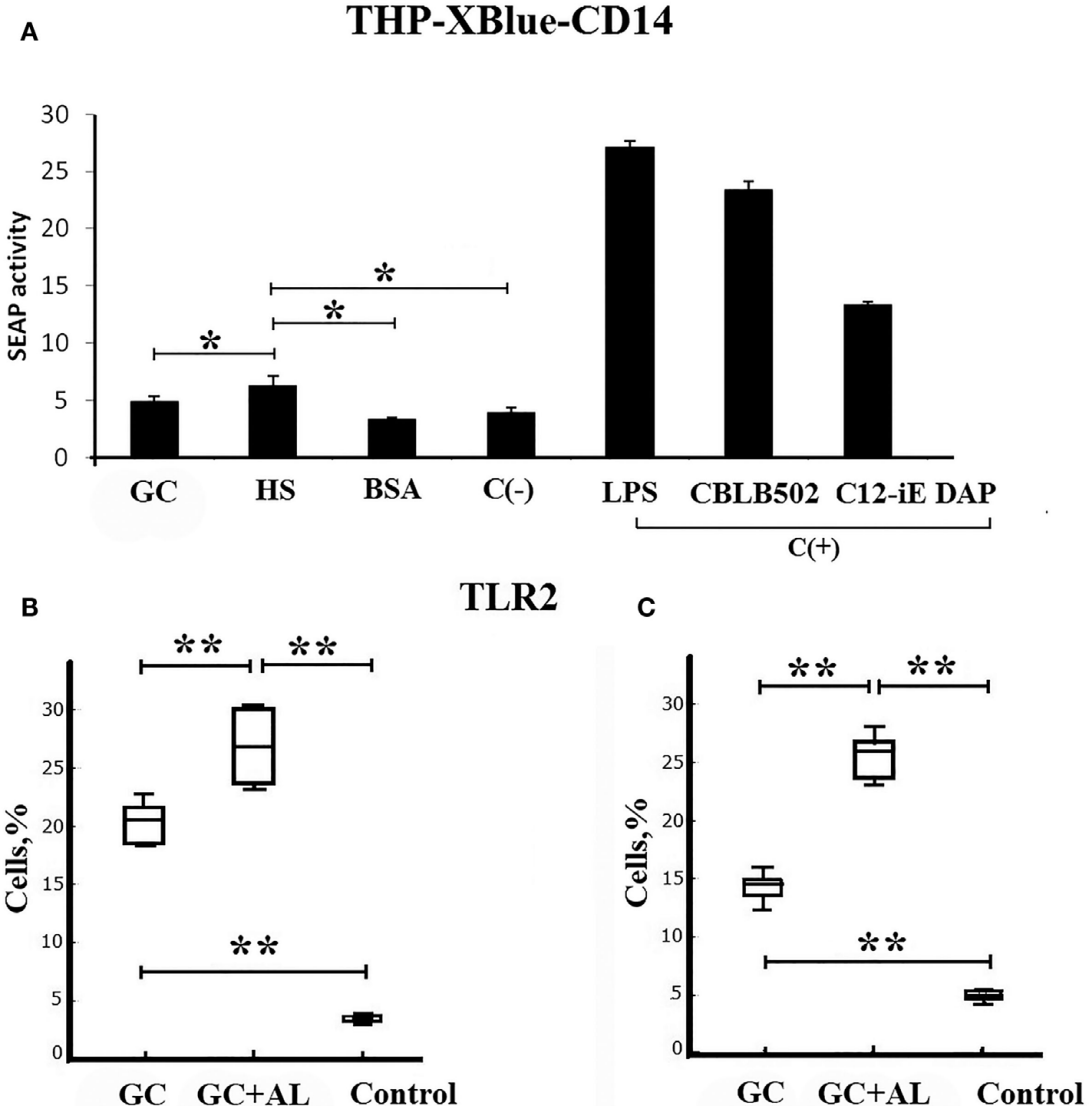
**Toll-like receptors**. **(A)** Direct ligand–receptor interaction. SEAP activity determined using the THP1-XBlue-CD14 human monocytic cell line expressing endogenous different types of pathogen-recognition receptors. In the SEAP assay test, HS, GC, and BSA were used at the same concentration (100 μg/mL). The negative control (C^−^) is PBS. The positive controls (C^+^) are LPS, CBLB502, and C12-iE-DAP, which are ligands for TLR4, TLR5, and NOD1, respectively. **(B)** Cell surface TLR2 molecules on the spleen mononuclear leukocytes (SML) on day 7 after the single intraperitoneal immunization of CBA mice (*n* = 6) with GC or GC + AL (10 μg of carbohydrate per mouse). Non-immunized mice (*n* = 6) served controls. **(C)** The same as above 7 days after the second immunization (*n* = 6). TLR2 levels on the SML were determined by flow cytometry. The results of the experiments are presented as box plots. The limits of the boxes represent the 25th and 75th percentiles of the results. The enclosed line represents the median (50th percentile), and the bars represent the 10th and 90th percentiles. The significance of differences between the groups was determined using the Mann–Whitney Rank Sum test. *P < 0.05.

Hexasaccharide enhanced SEAP activity compared to the negative control (*p* = 0.049535), GC (20 μg based on HS), and BSA (Figure 2A). LPS, CBLB 502, and C12-iE-DAP, which are ligands for TLR4, TLR5, and NOD1, respectively, and served as positive control for evaluation of THP1-XBlue-CD14 NF-kB reporter cells activation, increased SEAP activity when used at a concentration that was 100 times lower (1 μg/mL).

The study of TLR expression on mononuclear leukocytes from the spleens of mice immunized with GC and GC + AL demonstrated that the population of TLR2-expressing cells increased compared to the non-immunized control animals, served as a control on the seventh day after the first immunization (Figure 2B). After the second immunization, the level of TLR2-expressing cells remained constant and differed from the control (Figure 2C). GC + AL stimulated the population of TLR2-expressing cells after both the first and second immunization to a greater extent than GC (*p* = 0.009). There were no changes in the levels of TLR4-expressing cells (data not presented). The expression of TLR2 on spleen cells from immunized mice was not the outcome of a ligand–receptor interaction according to the data of SEAP cell assay.

### Dendritic Cells Phenotype and Cytokine Production

**Figure 3:**
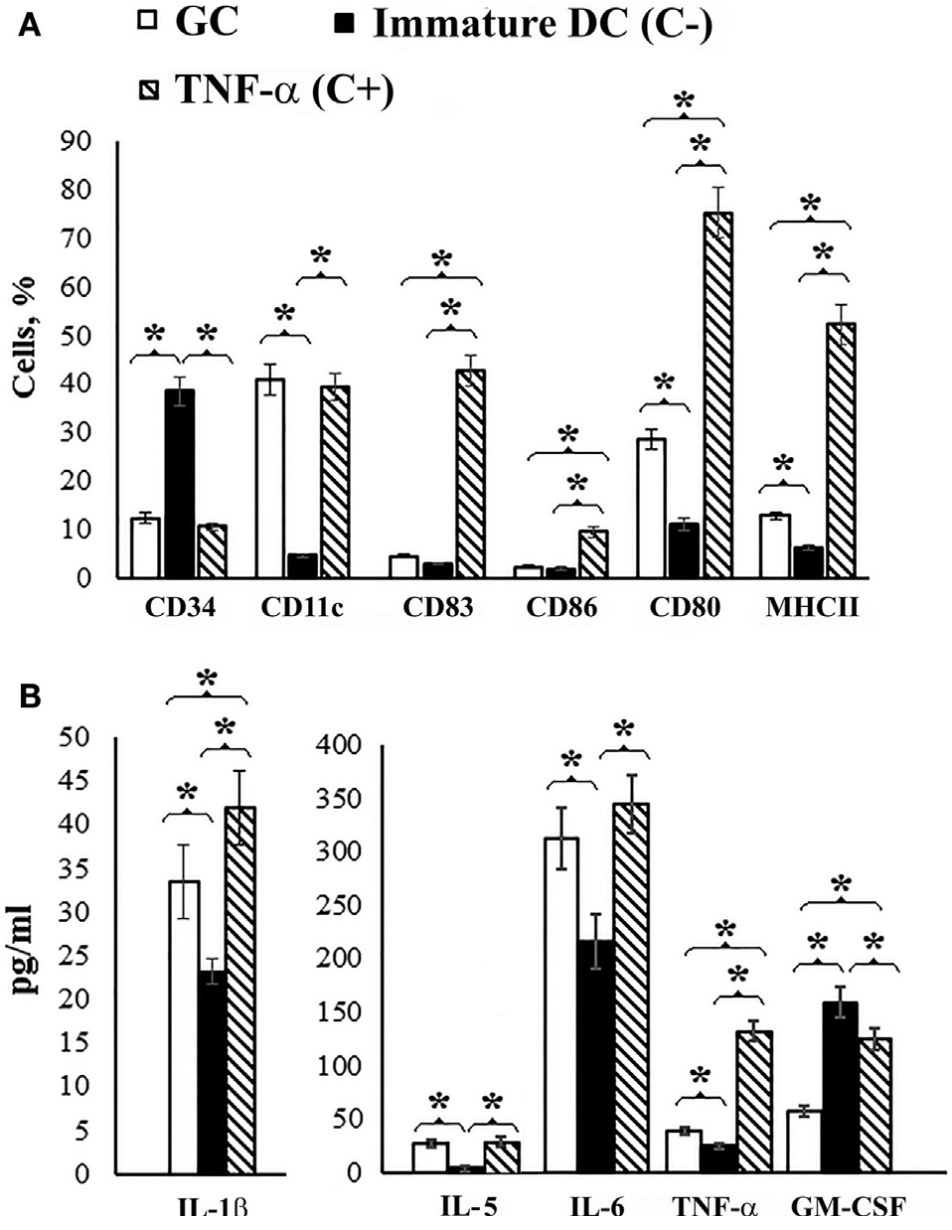
**Dendritic cell maturation**. Dendritic cell precursors obtained from mouse bone marrow were incubated with IL-4 and GM-CSF for 6 days. On day 7, GC was added to the cell culture in the concentration 50 μg/mL and 3 days after incubation determined cell surface molecular expression. Non-treated cells served as a negative control (C^−^). Cells incubated with TNFα served as positive controls (C^+^). **(A)** Cell surface molecules on the dendritic cells. CD34 is a molecule that is found on immature cells, CD11c is an adhesion molecule, CD80 and CD86 are costimulatory molecules, CD83 is a molecule of terminally differentiated dendritic cells, and MHCII is the major histocompatibility complex class II. The Abs that used included: FITC or PE-conjugated Abs against CD34 (clone RAM34, FITC), CD11c (clone HL3, FITC), CD83 (clone Michel-17, FITC), CD86 (clone PO3.1, PE), CD80 (clone 16-10A1, FITC), MHCII (I-EK, PE) (clone 14-4-45), all obtained from eBioscience (eBioscience Inc., San Diego, CA, USA). **(B)** Cytokine production in the culture medium of treated and non-treated cells. The levels of IFNγ, IL-2, IL-4, IL-10, and IL-17 did not differ from immature DC. Cell surface molecules on the dendritic cells and cytokine levels were determined by flow cytometry. The data are shown as the mean ± SD. The significance of differences between the groups was determined using the Mann–Whitney Rank Sum test. *P < 0.05.

The addition of GC (50 μg/mL) to the culture of immature DCs generated from mouse bone marrow resulted in a decrease in the level of non-differentiated CD34^+^ cells and an increase in the population of cells expressing the adhesion molecule CD11c, the costimulatory molecule CD80, and the antigen presentation molecule MHC class II compared to immature DC (*p* < 0.05) (Figure 3A). Thus, in the presence of GC, the phenotype of DCs corresponded to that of mature DCs (CD11c^+^, CD80^+^, MHC class II^+^); however, except for CD11c-expressing cells, their levels were lower than in the presence of TNFα (*p* < 0.05), which was used as a positive control (C^+^).

Under the action of GC, DCs secreted IL-1β, IL-6, and TNFα, into the culture medium with decreasing GM-CSF levels compared to immature DCs (C^−^) (*p* < 0.05) (Figure 3B). The levels of IFNγ, IL-2, IL-4, IL-10, and IL-17 did not differ from immature DC; however, the level of IL-5 was higher than the negative control.

### Cytokine Production in Mice

**Figure 4:**
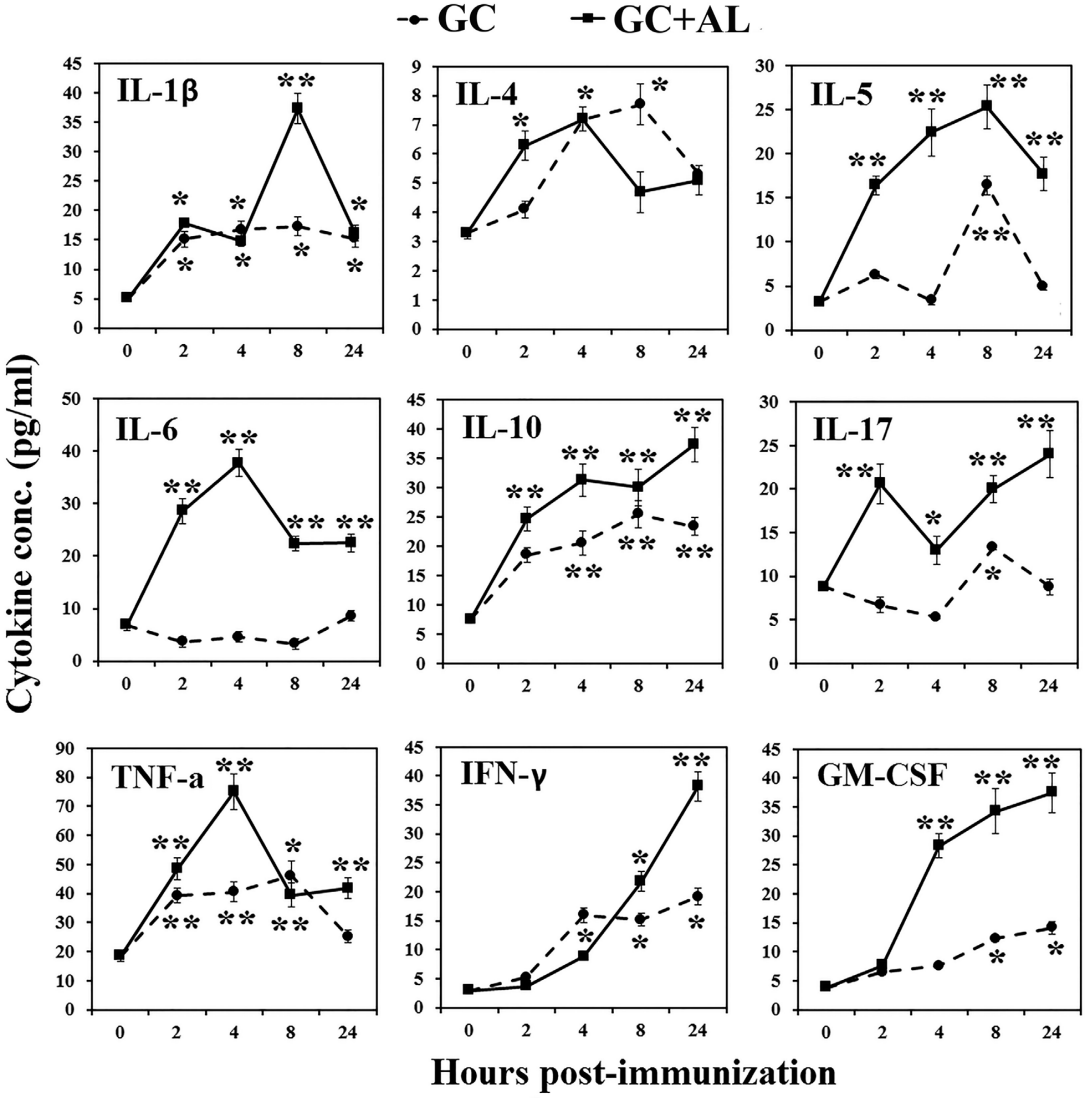
**Cytokine production in mice**. Mice CBA were immunized with GC or GC + AL (10 μg of carbohydrate per mouse). Serum was collected from mice (*n* = 6 for each time-point) at the indicated hours after immunization. The cytokine levels were determined by flow cytometry. IL-2 level did not increase in any of the observation periods (data not presented). The data are shown as the mean ± SD. *P < 0.05; **P < 0.01.

To evaluate cytokine production *in vivo*, GC or GC + AL was intraperitoneally administered to mice at a single dose of 10 μg based on the carbohydrate. Serum cytokine levels were determined before GC injection (hour 0) and 2, 4, 8, and 24 h after administration (Figure 4).

In response to immunization with GC, IL-1β increased at 2 h and remained at that level for 24 h (*p* < 0.05). The effect of GC + AL during 4 h was the same, but at 8 h, IL-1β transiently increased (*p* < 0.01). IL-2 did not increase in any of the observation periods (data not presented). In response to GC, IL-4 short-term increase was at 4 h (*p* < 0.05). GC + AL stimulated the production of IL-4 in the interval from 2 to 8 h (*p* < 0.05). IL-5 under the action of GC increased at 8 h (*p* < 0.01), whereas in response to GC + AL remained at a high level in the interval from 2 to 24 h (*p* < 0.01). IL-6 did not change in response to GC, whereas the injection of GC + AL increased its production in the interval from 2 to 24 h (*p* < 0.01). IL-10 increased after immunization with GC and GC + AL from 2 to 24 h (*p* < 0.01); however, GC + AL stimulated higher level of its production. IL-17 under the action of GC transiently increased at 8 h (*p* < 0.05) and in the response to GC + AL was in the higher level from 2 to 24 h (*p* < 0.01). TNFα in response to GC and GC + AL increased in the period from 2 to 24 h (*p* < 0.01), but under the action of GC + AL was a peak at 4 h (*p* < 0.01). IFNγ under the action of GC and GC + AL increased from 4 to 24 h (*p* < 0.05) GC + AL stimulated the highest production at 24 h (*p* < 0.01). GM-CSF in response to GC increased from 8 to 24 h, GC + AL led to higher its production from 4 to 24 h (*p* < 0.01).

### Glycoconjugates Absorbed and Unabsorbed on Aluminum Hydroxide Exert Different Effects on the Levels of T and B Lymphocytes

**Figure 5:**
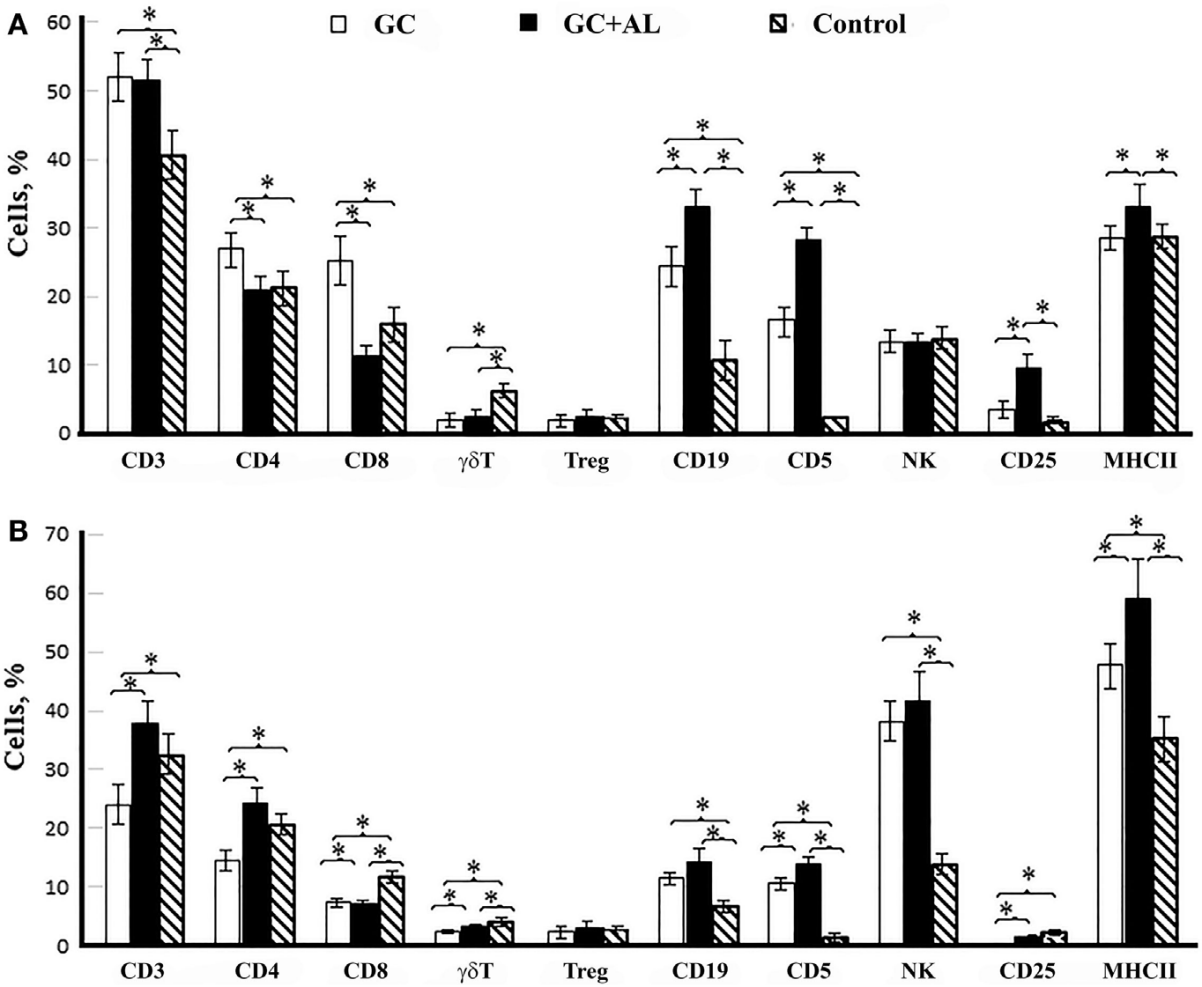
**Cell surface molecule expression by splenic mononuclear leukocytes**. CBA mice were immunized with GC or GC + AL (10 μg of carbohydrate per mouse). Non-immunized mice (*n* = 6) served as a control. T lymphocytes (CD3, CD4, and CD8); B lymphocytes (CD19, CD5); NK; CD25 is the IL-2 receptor on T cells and MHC class II were determined by flow cytometry. **(A)** 7 days after the first immunization (*n* = 6); CD4/CD8 ratio: GC – 1.08, GC + AL – 1.82. **(B)** 7 days (day 21) after the second immunization (*n* = 6); CD4/CD8 ratio: GC – 1.92, GC + AL – 3.36. Spleen cell suspensions were stained using antibodies specific for mouse CD3e-FITC (clone 145-2C11), CD4-FITC (clone GK1.5), CD8a-FITC (clone 53-6.7), γδT (clone γδ TCR-PE, eBioGL3), CD19-FITC (eBio1D3), CD5-PE (clone 53–7.3) NK.1.1 (clone PK136) CD25-PE (PC61.5) and MHCII-PE (I-EK) (clone 14-4-45) obtained from eBioscience or Biolegend (San Diego, CA, USA). Treg: Alexa Fluor 405-conjugated anti-mouse CD4 (clone RM4-5) was obtained from Caltag Laboratories. Staining with anti-FoxP3-FITC conjugated Ab (clone FJK-16s) was performed according to the manufacturer’s protocol (eBioscience). The data are shown as the mean ± SD. Mann–Whitney Rank Sum test. *P < 0.05.

The immunization of mice with GC in the presence or absence of aluminum hydroxide elicited differences in the surface molecule expression levels on mononuclear leukocytes from mouse spleens depending on the presence of an adjuvant and immunization multiplicity (Figure 5).

A single immunization of mice with GC increased the number of T lymphocytes expressing surface molecules CD4 (T helper) and CD8 (*p* < 0.05 vs. control and GC + AL) as well as B cells expressing CD19 and CD5 markers (*p* < 0.05 vs. control) (Figure 5A). GC + AL exerted no effect on the content of CD4^+^ and CD8^+^ T cells but favored, to a greater extent, an increase in the number of B cells (CD19^+^, CD5^+^) and T cells that were positive for the IL-2 receptor (CD25^+^) and the number of cells expressing major histocompatibility complex (MHC) class II molecules (*p* < 0.05 vs. control and GC). Both GCs decreased the number of γδT lymphocytes but exerted no effect on the number of regulatory T lymphocytes (Treg) and natural killers (NK).

After the second immunization of mice with GCs, their effect on the cell-mediated immune response changed substantially (Figure 5B). The injection of GC decreased the CD4^+^ T lymphocyte numbers (*p* < 0.05 vs. control), whereas GC + AL exerted no effect on the number of T helper cells. Both GCs resulted in a decrease in the level of T cells with the expression of CD8^+^ T cell and γδT cell surface molecules (*p* < 0.05 vs. control). The CD4/CD8 ratio increased in the presence of aluminum hydroxide due to a decrease in CD8^+^ T cell numbers (for GC, 1.92, whereas for GC + Al, 3.36). The number of B cells (CD19^+^, CD5^+^) remained enhanced (*p* < 0.05 vs. control) as a response to the repeated injection of each GC, and the number of cells expressing MHC class II was highest after immunization with GC + AL (*p* < 0.05 vs. control and GC). The number of NK cells increased under the action of GC and GC + AL.

### Glycoconjugate Absorbed on Aluminum Hydroxide Induces a Long-term IgG-Antibody Response to PS and Immunological Memory

**Figure 6:**
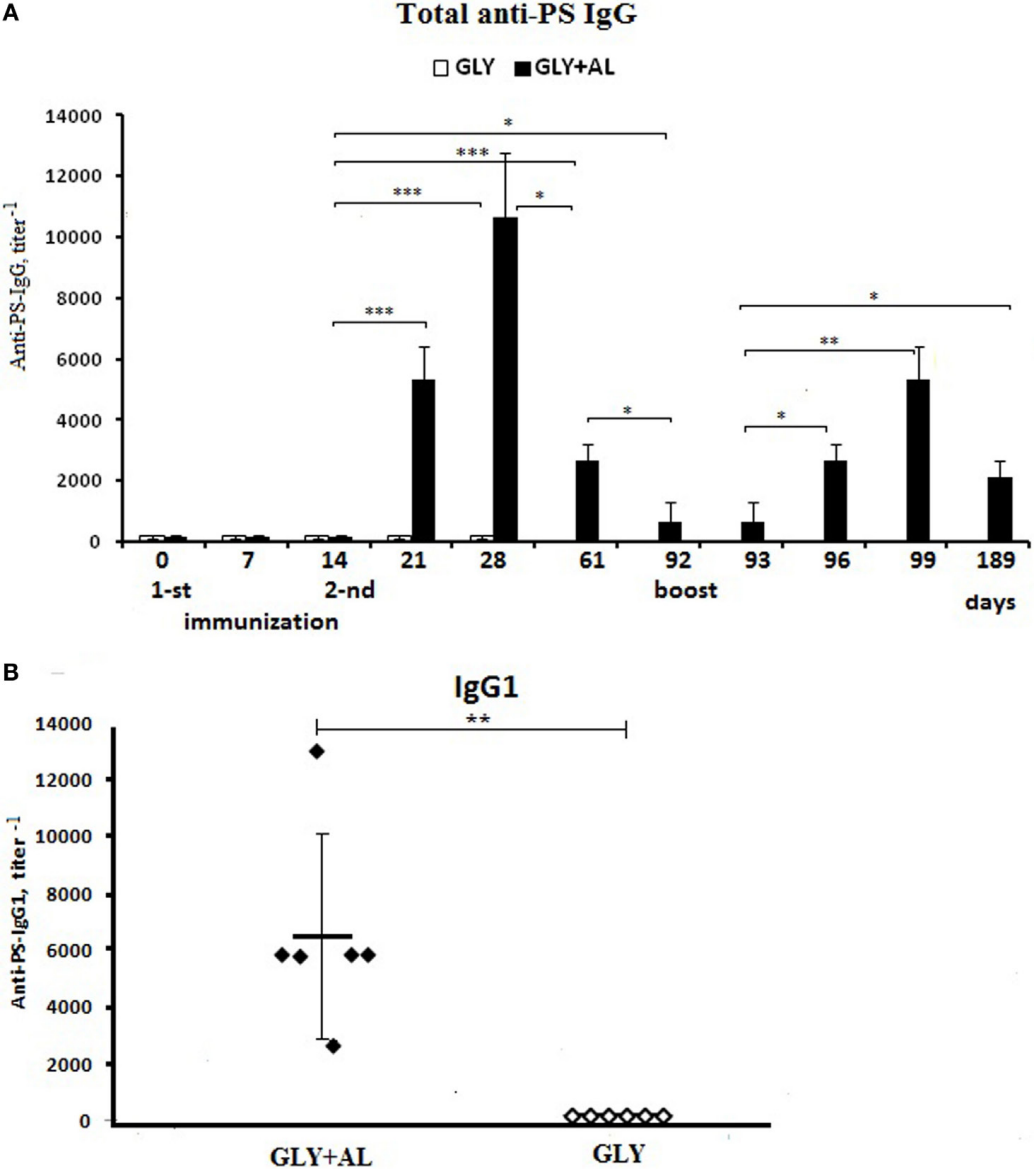
**Antibody production**. CBA mice were immunized intraperitoneally with GC (white bars) or GC + AL (black bars) (10 μg of carbohydrate per mouse) at days 0 and 14 and were boosted on day 92 with the same dose of GC + AL. **(A)** Anti-PS IgG. Serum samples were collected at the indicated time intervals from mice (*n* = 6) and tested by ELISA using plates coated with PS. Antibody titers were expressed as the dilution giving twice the OD obtained for mice on the day 0. The data are shown as the mean ± SEM. **(B)** IgG1. Individual titers of the anti-PS antibodies on day 28 (day 14 after the second immunization) in BALB/c mice vaccinated with a dose of 10 μg in the presence (black points) or absence (white points) of aluminum hydroxide. Each group contained six mice. The data are shown as individual data points and mean ± SD. One-way ANOVA and Tukey posttest, *P < 0.05; **P < 0.01; ***P < 0.001.

Mouse serum PS *S. pneumoniae* type 14-specific IgG levels were measured before and at different time intervals after the two intraperitoneal immunizations of mice with GC and GC + AL followed by a booster immunization with GC + AL on day 92 (Figure 6).

The first immunization with GC or GC + AL at a single dose of 10 μg based on carbohydrate-specific IgG levels did not differ from non-immunized mice (day 0) on days 7 and 14 (Figure 6A). One week (day 21) following the second immunization with GC + AL, IgG levels increased (*p* = 0.000177) and continued to increase until day 28, whereas GC alone did not induce antibody production, which was also demonstrated by testing for IgG1 in individual mouse sera (Figure 6B).

IgG levels after GC + AL immunization remained high until day 61 (47 days after the second immunization) compared to the initial level (days 0–14) (*p* = 0.000205). On day 92, IgG levels decreased but remained higher than the initial values (*p* = 0.011659). The individual titers of IgG1 14 days (day 28) after the second GC + AL immunization are displayed in Figure 6B. The booster immunization GC + AL using GC (dose 10 μg based on carbohydrate) was performed on day 92 on a background of low IgG levels (Figure 6A). One day later (day 93), the IgG levels remained at the initial level, but 4 days later (day 96), the IgG titers increased (*p* = 0.012195) and continued to increase on the seventh day (day 99) (*p* = 0.01148). The rapid generation of IgG indicates the formation of immunological memory to PS. The activation of immunological memory resulted in a prolonged antibody response within 97 days after a booster GC + AL injection (day 189), and the anti-PS IgG titers remained higher than before revaccination (*p* = 0.031494).

The IgG2a, IgGb, and IgG3 levels after GC + AL immunization did not substantially increase (the data are not presented).

## Discussion

Certain success was achieved with the development of synthetic vaccines against a number of infectious diseases. Vaccines against *Haemophilus influenzae* type b (33), meningococcus group C (34), *Staphylococcus* (35), and other socially significant infections are being developed.

The development of synthetic pneumococcal vaccines is aimed at searching for immunogenic oligosaccharides that induce, being components of GCs, the production of protective antibodies to capsular PSs of etiologically significant serotypes of pneumococcus, including *S. pneumoniae* type 14 (5). The conjugates of carrier proteins with some synthetic oligosaccharides related to fragments of capsular PSs of various *S. pneumoniae* serotypes were studied as potential vaccines (29).

The production of IgG antibodies to HS conjugated with BSA required the presence of an adjuvant and two immunizations, after which the high level of antibodies to capsular PS and the formation of immunological memory in response to revaccination were observed. Aluminum hydroxide was used as an adjuvant. The highest level of antibodies 1:6400–1:12800 in response to HS conjugated with BSA and absorbed on aluminum hydroxide was revealed in 2 weeks after double immunization. According to our data, the titer of IgG antibodies to Prevenar-13 with aluminum phosphate, as adjuvant, after double intraperitoneal immunization of mice with a dose of 1.1 μg/mouse for PS *S. pneumoniae* type 14 was 1:800–1:1600 in ELISA (data not presented).

It is known that the immune response starts from the activation of the receptor apparatus of APCs. There are data indicating that 23-valent pneumococcal PS vaccine Pneumovax-23 stimulated the expression of TLR2 and TLR4 and that pneumococcal conjugate vaccine Prevnar activated TLR2, which resulted in an increase in the anti-PS IgG response in mice predominantly through IgG2a, IgG2b, and IgG3 rather than IgG1. In the authors’ opinion, TLR activation occurred under the action of bacterial impurities present in commercial preparations (23). However, in a test of transgenic *TLR2^−/−^* mice with a defect in the TLR2 gene expression, the production of specific IgM under the action of PS *S. pneumoniae* types 3 and 14 was lower than that of wild-type mice (24). It is notable that TLR2 expression levels were not determined.

We showed an increase in the number of cells with TLR2 expression on mononuclear leukocytes of the spleens of mice immunized by synthetic HS conjugated with BSA. The activation of TLRs was non-specific and likely occurred under the action of cytokines and other “danger signals,” which was confirmed by a minor (compared to positive controls) increase in the SEAP activity of THP1-XBlue™-CD14 cells affected by HS. Thus, the expression of TLR2 on spleen cells from immunized mice was not the outcome of a ligand-receptor interaction according to the data of SEAP cell assay.

The activation of antigen-specific T cells is controlled by signal 1 (TCR) and signal 2 (costimulatory molecules). Signal 3 is believed to exist in the form of pro-inflammatory cytokines produced by APCs, and they directly affect T cells (36). TLRs expressed on APCs are responsible for the generation of signal 2 and signal 3 (37). During vaccination, aluminum hydroxide has also been shown to generate signals 2 and 3 (38), i.e., it can compensate for insufficient TLR activation and cytokine production to induce T cell activation.

Glycoconjugate induced the maturation of DCs generated from mouse bone marrow *in vitro*. Mature CD11c^+^, CD80^+^, and MHC class II^+^ DCs secreted IL-1β, IL-6, and TNFα into the culture medium. However, surface molecule expression and cytokine production were lower compared to the positive control (TNFα). The level of IL-1β production was higher than that of the positive control only if the conjugate absorbed on the aluminum hydroxide was added into the DC culture medium.

A wide spectrum of serum cytokines was determined in mice after a single administration of GC absorbed on aluminum hydroxide, which suggests the activation of various cells of the immune system. GC without an adjuvant resulted in the prolonged production (from 2 to 24 h) of IL-1β, IL-10, and TNFα alone, GM-CSF (from 4 to 24 h), and IFNγ (from 8 to 24 h). In this protocol, the levels of IL-6 which is involved in B lymphocyte differentiation, antibody-forming cell maturation, and immunoglobulin production remained unchanged, whereas, IL-4, IL-5, IL-17, cytokines increased in a transitory manner and to a lesser extent after immunization with the absorbed preparation. The preparation absorbed on aluminum hydroxide stimulated an increased production of all studied serum cytokines: IL-1β, IL-4, IL-6, IL-10, IL-17, GM-CSF, IFNγ, and TNFα. Transient increase of IL-1β and TNFα was observed at 8 and 4 h, respectively. In the previous study, we revealed that sera obtained at 4 h after the single immunization of mice with GC without aluminum hydroxide did not possess bactericidal activity and those obtained at 24 h only weakly promoted phagocytosis of the live culture of heterologous pathogen (*Staphylococcus aureus*) by leukocytes. GC absorbed on aluminum hydroxide increased bactericidal activity of peripheral blood leukocytes of mice obtained at 4 h and more intensively at 24 h after the single immunization (39).

After the second immunization, the GC absorbed on aluminum hydroxide resulted in the formation of IgG antibodies to capsular PS. In the previous studies, it was shown that the hexasaccharide conjugate adsorbed on aluminum hydroxide protected immunized mice from *S. pneumoniae* serotype 14 (39). Without aluminum hydroxide, the GC did not possess the protective properties (39). In this period, against the background of normal CD4^+^ T helper cell numbers, the suppressor effect of CD8^+^ T lymphocytes decreased, the expression of the IL-2 receptor (CD25^+^) on T lymphocytes increased, the number of B lymphocytes increased, and the expression of MHC class II molecules on mononuclear leukocytes from the spleen increased. IgG1 was predominantly formed, which assumes the polarization of the immune response occurs mainly *via* the Th2 route. In addition to its activating effect on Th2 cells, the absorbed GC activated Th1 cells, producing IFNγ and Th17 cells, leading to IL-17, which is involved in the protection from extracellular bacteria, including *S. pneumoniae* (40). The predominant direction of the immune response *via* the Th2 route is probably caused by the presence of aluminum hydroxide (41).

## Conclusion

We determined the key stages of the activation of the innate and adaptive immune responses in mice by a BSA conjugate of a synthetic HS that was related to the fragment of the capsular PS of *S. pneumoniae* type 14. The revealed specific features of GC action on the activation of innate and adaptive immunity make it possible to enhance their immunogenic properties and to optimize the design of synthetic vaccines. It may also be promising to search for efficient adjuvants for oligosaccharide-conjugated vaccines.

## Author Contributions

NA – planning study, assessment of the immunophenotype of dendritic cells and mononuclear leukocytes of mice, statistical analysis of data. EK – a study on the duration of antibody response in mice. Evaluation of immune response after booster administration to mice, a conjugate with a synthetic hexasaccharide. EA – obtaining mature dendritic cells, evaluating the level of cytokines in the supernatant of dendritic cells and in the sera of mice vaccinated with conjugated hexasaccharide, adsorbed on aluminum hydroxide. NE – summarizing the results, comparison with the data of contemporary literature, statistical analysis of the results of the study. DL – estimation of the direct ligand-receptor interaction of the hexasaccharide, the glycoconjugate and BSA was with THP1-XBlue™-CD14 cells derived from the THP1-XBlue™ cell line of human monocytes expressing different pathogen-recognizing receptors, including TLRs, and NOD-like receptors. MG – characterization of the capsular polysaccharide of *S. pneumoniae* type 14 and assessment of its ability to recognize antibodies to the conjugated hexasaccharide in ELISA. Elena V. Sukhova – synthesis of the hexasaccharide fragment of the capsular polysaccharide of *S. pneumoniae*. Evaluation of the immunological activity of the conjugated hexasaccharide in ELISA. Dmitry V. Yashunsky – synthesis and characterization of the hexasaccharide.

## Conflict of Interest Statement

The authors declare that the research was conducted in the absence of any commercial or financial relationships that could be construed as potential conflict of interest.

## Abbreviations

BSA, bovine serum albumin; CD, cluster of differentiation; DC, dendritic cell; ELISA, enzyme-linked immunosorbent assay; GC, glycoconjugate; GC + AL, glycoconjugate with aluminium hydroxide; GM-CSF, granulocyte-macrophage colony-stimulating factor; IFN, interferon; IL, interleukin; MALDI-TOF, matrix-assisted laser desorption/ionization time-of-flight; MHC class II, major histocompatibility complex class II; PS, capsular polysaccharide; SEAP, secreted alkaline phosphatase; Th, T helper; TLR, toll-like receptor; TNF, tumor necrosis factor.
